# Advantages of Cultivating Medical Physiognomy

**Published:** 1843-07

**Authors:** 


*Advantages of Cultivating Medical Physiognomy.*—The advantages of an acute exercise of the perceptive powers, although strikingly exemplified in the practice of many eminent men, have, in general, been little inculcated by medical writers and teachers. We hear, indeed of the *tactus eruditus*, and the use of the ear in auscultation is now universally admitted as an important aid to diagnosis; the *decubitus*, also, and a few other points relating to attitude, are commented upon by medical writers. But there is a peculiar learning belonging to the *eye* and *ear* which claims the most careful attention, although hitherto but little appreciated. It is well known how skilfully some men can "find the mind's construction in the face," and detect a latent meaning in the simplest gesture or the slightest variety of intonation. This constitutes *physiognomy*, in the widest sense of the term, and every observant person must know that physiognomy has a foundation in nature, though Lavater and others did not succeed in reducing it to a science. But has *disease* no physiognomy? Do morbid actions imprint on the countenance no marks, and communicate to the voice, gesture, and manner, no peculiarities by which the practised eye and ear may detect the presence of those morbid actions, and trace their changes and transitions? Assuredly disease *has* a physiognomy, and happy is the practitioner who is gifted with the power of discerning those minute shades of expression which are inappreciable to obtuse senses; for in this very power resides a great part of that almost magical precision in diagnosis and prognosis by which some individuals have been so remarkably distinguished. The reality of medical physiognomy cannot be better illustrated than by a very common example,—that of an affectionate mother watching her sick child, in which case, excited feelings and fixed attention give an acuteness to the perceptions that more than supplies the place of scientific culture. How often do we hear a mother, so circumstanced, declare that her child is better or worse, although we can, at the time, perceive no change in the symptoms? Yet the mother is almost invariably correct in her prognosis, and a change in the symptoms speedily follows.

The study of medical physiognomy is, for several reasons, one of extreme difficulty. First, because the greater part of mankind are probably not endowed by nature with æsthetic powers susceptible of the requisite degree of cultivation; secondly, because the finest senses are liable, perhaps the most liable, to be led astray by fancy; and as the most skilful microscopical observers occasionally see that which is invisible, and the most skilful stethoscopists full often hear that which is inaudible, so the most acute medical physiognomist is apt to be misled by the like *ignes fatui* of the fancy, and the study is one in which a very strict guard should be kept upon that treacherous faculty; thirdly, because if an individual attain a high degree of skill in medical physiognomy, that skill is, in a very small degree, communicable to others—the delicate though vivid impressions, of which his own highly cultivated senses render him susceptible, being, for the most part, incapable of explanation in language. Hence knowledge on such a subject can never become cumulative to a great extent, and each individual has little to draw upon excepting his own powers of original observation. The general outlines of the subject, however, admit of being traced in a manner intelligible to all, and it is much to be regretted that the physiognomy of disease is not generally recognised and taught as part of the study of practical medicine. Here, as in many other instances, the penetrating mind and vast genius of HIPPOCRATES have anticipated a more advanced era of medicine than we have even yet arrived at. His celebrated description of the countenance of approaching death is familiarly known; throughout his writings we find evidences that his external senses were constantly vigilant to aid his diagnosis and prognosis, and a greater number of physiognomical observations on diseases are to be found in his works than those of any succeeding author; although it should be remarked of the ancient Greek physicians generally, that they cultivated the æsthetic powers with reference to medicine much more diligently than the moderns, and semeiology was consequently in a more advanced state among them, proportionally to the other branches of medical science.—*London Lancet, April* 1, 1843.

